# Acknowledgement of manuscript reviewers 2015

**DOI:** 10.1186/s12955-016-0435-5

**Published:** 2016-02-25

**Authors:** Holger Schunemann, Feng Xie

**Affiliations:** McMaster University, Hamilton, Canada

## Abstract

The editors of *Health and Quality of Life Outcomes* would like to thank all our reviewers who have contributed to the journal in Volume 13 (2015).

**A Klasnja**

USA

**Aaron Reeves**

UK

**Abbas Sheikhtaheri**

Iran

**Abebaw Mengistu Yohannes**

UK

**Abebaw Mengistu Yohannes**

UK

**Adelberto Loyola-Sanchez**

Mexico

**Aderonke Akinpelu**

USA

**Aditya Gupta**

India

**Adomas Bunevicius**

USA

**Aja Louise Murray**

UK

**Albert Wu**

USA

**Ali Montazeri**

Iran

**Aliasghar Ahmad Kiadaliri**

Sweden

**Alicia Kunin-Batson**

USA

**Alina Andras**

UK

**Amanuel Tesfay**

USA

**Amélie Anota**

France

**Amir H Pakpour**

Iran

**An De Groef**

Belgium

**Ana Flavia Granville-Garcia**

USA

**Ana-Maria Iosif**

USA

**Ana-Maria Vranceanu**

USA

**Anastasios Athanasopoulos**

USA

**Andrea Acevedo**

USA

**Andrea Fuhs**

Germany

**Andrea Marcellusi**

Italy

**Andreas Fors**

Sweden

**Andreas Hinz**

Germany

**Andreas Schmitt**

Denmark

**Andrew Lloyd**

USA

**Andrew Moore**

UK

**Andrew Murnane**

USA

**Anette Bygum**

Denmark

**Angela Fong**

Canada

**Angelo Lavano**

Italy

**Aniela Krezel**

UK

**Anja Heilmann**

UK

**Ann Macaskill**

UK

**Anna Myléus**

Sweden

**Anne Kouvonen**

Finland

**Anne Peasey**

UK

**Anne-Françoise Spinoit**

Belgium

**Antonella Gritti**

Italy

**Anurag Patidar**

USA

**Arnaud Fauconnier**

France

**Arndt Buessing**

Germany

**Arthur Greil**

USA

**Arthur Mesas**

USA

**Ashley Smith**

USA

**Astri Ferdiana**

Netherlands

**Atsushi Tanaka**

Japan

**Augusta Silveira**

Portugal

**Ayse Kuspinar**

Canada

**Azlina Yusuf**

Malaysia

**B Jorner**

Denmark

**Barbara Bucki**

France

**Barbara Conner-Spady**

USA

**Barrie Cassileth**

USA

**Bb Hansen**

Denmark

**Bellinda King-Kallimanis**

USA

**Ben Ko**

UK

**Benedicte Dubois**

Belgium

**Benjamin Brucker**

USA

**Benjamin Craig**

USA

**Benjamin Gierk**

Germany

**Benjamin Leiby**

USA

**Bente Vederhus**

USA

**Bernd Puschner**

Germany

**Bianca Notario**

Spain

**Bianchi Elisabetta**

Italy

**Birte Østergaard**

Denmark

**Boon How Chew**

Malaysia

**Brendan Mulhern**

UK

**Brendon Stubb**

UK

**Brent Schacter**

Canada

**Brett Doble**

UK

**Brett Vaughan**

Australia

**Brian Marx**

USA

**Brian Westerberg**

Canada

**Brittany Rosenbloom**

Canada

**Bruno Damasio**

Mexico

**Bryan Dirks**

USA

**B T Howrey**

USA

**Burel Goodin**

USA

**C Høyer**

Denmark

**C Ramírez-Maestre**

Spain

**C Wang**

China

**Caitlyn Solem**

USA

**Carlo Diclemente**

USA

**Carlos Suso-Ribera**

Spain

**Carlos Wong**

Hong Kong

**Carlos Zubaran**

Australia

**Carmen Logie**

Canada

**Carmen Rodriguez Blazquez**

Spain

**Caroline Bascoul-Mollevi**

Germany

**Caroline Jenkinson**

UK

**Casey Peiris**

USA

**Catherine Draper**

South Africa

**Chad Gwaltney**

USA

**Charles Emlet**

USA

**Chiara Scaratti**

Italy

**Ching-Lin Hsieh**

Taiwan

**Chong Guan Ng**

Malaysia

**Chris Gibbons**

UK

**Chris Markham**

UK

**Christina Aggar**

Australia

**Christina Peterson**

Sweden

**Christine Blome**

Germany

**Christine Fu**

USA

**Christine Mcdonough**

USA

**Christopher Brennan-Jones**

UK

**Chun Fan Lee**

Singapore

**Claides Abegg**

Brazil

**Claire Gudex**

USA

**Claire Snyder**

USA

**Cm Verolet**

Switzerland

**Colin Kennedy**

UK

**Colm Mcalinden**

UK

**Corneel Coens**

Belgium

**Cornelia H M Van Den Ende**

Netherlands

**Cristina Bostan**

Switzerland

**C Y Hsiao**

Taiwan

**D Barthel**

Germany

**D Deutscher**

USA

**Dallas Swendeman**

USA

**Damian Griffin**

UK

**Dan Kaye**

Uganda

**Daniel Costa**

Australia

**Daniel E Jimenez**

USA

**Daniel Frendl**

USA

**Daniel Heinl**

Germany

**Daniel Joseph Cher**

USA

**Daniel Reissmann**

Germany

**David Andrae**

USA

**David Brennan**

Australia

**David Cassiman**

Belgium

**David Cella**

USA

**David Christiansen**

Denmark

**David Ellard**

UK

**David Eton**

USA

**David Morley**

UK

**David Rehkopf**

USA

**David Tw Yau**

USA

**David Whiteley**

USA

**Deanna Wathington**

USA

**Deirdre Gartland**

Australia

**Denise Duijster**

Netherlands

**Denise Hallfors**

USA

**Denise Kuster**

Germany

**Derek Tang**

USA

**Diogo Costa**

Angola

**Domenico Giacco**

UK

**Dominik Golicki**

Poland

**Dong Wook Shin**

Korea, Republic of

**Donna Evon**

USA

**A F Donneau**

Belgium

**Douglas Nguyen**

USA

**E Heintz**

Sweden

**E Poku**

UK

**Eduardo Bernabé**

UK

**Eero Haapala**

USA

**Elise Lev**

USA

**Elisio Costa**

Portugal

**Elizabeth Van De Graaf**

Netherlands

**Ellen Meltzer**

USA

**Emel Bulcun**

USA

**Emily Finne**

Germany

**Emma Frew**

UK

**Emma L Patchick**

UK

**Eng Teo**

USA

**Enrique Coss-Adame**

USA

**Enzo Ballatori**

Italy

**Erla Bjornsdottir**

USA

**Ernesto Jaramillo**

USA

**Esther Lopez**

Spain

**Estrella Romero**

Spain

**Eva Penelo**

Spain

**Eva Segura-Orti**

Spain

**Evan Atlantis**

Australia

**Ewa Rodakowska**

Poland

**Fabiane Rossi Dos Santos Grincenkov**

USA

**Fabio Efficace**

Italy

**Fábio Mialhe**

Brazil

**Fatemeh Bazarganipour**

Iran

**Faterneh Asgarian**

USA

**Femida Handy**

USA

**Fergal Grace**

UK

**Fernando Colugnati**

USA

**Fernando Rodríguez-Artalejo**

Spain

**Fernando Torrente**

Argentina

**Fidel Lopez-Espuela**

Spain

**Filippo Graziani**

Italy

**Finbarr Allen**

Ireland

**Fiona Jones**

UK

**Florence Wong**

China

**Folake Lawal**

Korea, Republic of

**Francesca Chiesi**

Italy

**Francesco Cottone**

Italy

**Francois Dionne**

France

**Frank Modersitzki**

USA

**G E Theodoropoulos**

Greece

**G Krueskem**

Germany

**Gabriel Rebick**

USA

**Gabriella Barreto**

Brazil

**Gabrielle Britton**

Panama

**George Wong**

Hong Kong

**Ghufran Ahmed Jassim**

Bahrain

**Giancarlo Lucchetti**

Brazil

**Giorgio Bertolotti**

Italy

**Giovanni Battista Marcon**

Italy

**Giovanni Losco**

New Zealand

**Giulia Cavrini**

Italy

**Giulio Marchesini**

Italy

**Giuseppe Scaletta**

Italy

**Gopalakrishnan Netuveli**

UK

**Grace Su Yin**

Singapore

**Grace Yang**

Singapore

**Grant Searchfield**

New Zealand

**Gretchen A Good**

New Zealand

**Gudrun Rohde**

Norway

**Gul Mete Civelek**

Turkey

**Guus Luijben**

Netherlands

**Gwendolyn Janssen**

USA

**H Wang**

China

**Hamid Baradaran**

Iran

**Hamideh Salimzadeh**

Iran

**Hana Vonkova**

Czech Republic

**Hanna Grol-Prokopczyk**

USA

**Harald Bailer**

Germany

**H C Hsu**

Taiwan

**Helena García-Llana**

Spain

**Henrique Mansur**

Brazil

**Hillary Broder**

USA

**H J Baumann**

USA

**H K Sut**

Korea, Republic of

**Howard Vernon**

Canada

**Hueymin Wu**

Taiwan

**Hye-Young Kang**

Korea, Republic of

**I Schafer**

Germany

**Iaria Baiardini**

Italy

**Ilse Kryspin-Exner**

Austria

**Imad Maatouk**

Germany

**Ines Buchholz**

Germany

**Ira Sierwald**

Germany

**Ira Tromifova**

Canada

**Irene Teo**

Singapore

**Isaac Prilleltensky**

USA

**Ishveen Chopra**

USA

**Ivana Tadic**

Serbia and Montenegro

**J Beaumont**

USA

**J Lindo**

Jamaica

**J M Blazeby**

UK

**J White**

UK

**J Whitty**

Australia

**J A Spertus**

USA

**Jacek Kopec**

Canada

**Jakob Bjorner**

Denmark

**Jakob Bjørner**

USA

**James Danckert**

Canada

**James Fitzgerald**

USA

**James Twiss**

UK

**Jameson Hirsch**

USA

**Jan Koetsenruijter**

Netherlands

**Jana Wardian**

USA

**Jane A Buxton**

Canada

**Jane Davies**

Australia

**Janelle Yorke**

UK

**Janet Rosenbaum**

USA

**Janine Archer**

UK

**Javier Ballesteros**

Spain

**Javier Montero**

Spain

**Javier Rejas**

Spain

**Jazmin Brown-Iannuzzi**

USA

**Je Lieu**

USA

**Jennifer L Seddon**

UK

**Jennifer Petkovic**

Canada

**Jennifer Read**

UK

**Jenny Abanto**

Brazil

**Jessie Bakker**

USA

**Jessie Porter**

UK

**Jiansheng Li**

China

**Jill Stewart**

USA

**Jing Qian**

USA

**Jiun-Hau Huang**

Taiwan

**Jiyeon Lee**

Korea, Republic of

**Jj Lundy**

USA

**Jm Morys**

Poland

**Joana Ramos-Jorge**

Brazil

**Joana Salifu Yendork**

South Africa

**Joanne Haviland**

UK

**John A Maluccio**

USA

**John D Grainger**

UK

**John Eastwood**

Canada

**John Horwood**

New Zealand

**John Salsman**

USA

**John Whited**

USA

**John Winkelman**

USA

**Jonathon Holmes**

USA

**Jordi Alonso**

Spain

**Joseph M Herman**

USA

**Joshua Pink**

UK

**J P Barile**

USA

**Jr-Rung Lin**

Taiwan

**Juan L Garrido-Castro**

Spain

**Juan Manuel Ramos Goñi**

Spain

**Juanita A Haagsma**

Netherlands

**Judith Van-Der-Waerden**

France

**Julia Liew**

USA

**Julia Louw**

South Africa

**Julia Mcquillan**

USA

**Juliana Boza**

Brazil

**Julie Halverson**

USA

**Julie Ratcliffe**

Australia

**Julie Ryan**

USA

**Julius Burkauskas**

Italy

**Jun Aida**

Japan

**Jun-Hao Pan**

China

**Jyoti Khadka**

Australia

**K Fackrell**

UK

**K Nicholson**

Canada

**K Weitkamp**

Germany

**Kameran Ismail**

Iraq

**Kamlesh Singh**

India

**Karen Hirschman**

USA

**Karen Horridge**

USA

**Karen Kuhlthau**

USA

**Karine Baumstarck**

France

**Katashi Okoshi**

Brazil

**Kate Lycet**

Australia

**Kate M Bennett**

UK

**Katharina S Sunnerhagen**

USA

**Kathleen Yost**

USA

**Kavitha Nutakki**

USA

**K C Chang**

Taiwan

**Ke Li Yin**

China

**Kelly Shaffer**

USA

**Kerim Beseoglu**

Germany

**Kevin Friedman**

USA

**K F Hoth**

USA

**Kim Kiely**

Australia

**Kim Zlomke**

USA

**Kimiaki Utsugisawa**

Japan

**K M Van Leeuwen**

Netherlands

**Konrad Pesudovs**

Australia

**Kristen Jastrowski Mano**

USA

**Kristin Haraldstad**

Norway

**Kristin Thorborg**

Denmark

**Kristofer Bandayrel**

Canada

**K S Lee**

Korea, Republic Of

**Kyung Ho Park**

Korea, Republic Of

**L Hayes**

Australia

**L Schoonhoven**

Netherlands

**Lacey Sischo**

USA

**Lan Yu**

USA

**Lars G Johnsen**

Norway

**Laura Pinheiro**

USA

**Laura Platinga**

USA

**Laurent Boyer**

Italy

**Leandro Rezende**

Brazil

**Lei Hum Wee**

Malaysia

**Leila Jahangriry**

Iran

**Leili Salehi**

Iran

**Lena Spangenberg**

Germany

**Leslie J Hinyard**

USA

**Li Zhigang**

USA

**Lilian Jans-Beken**

Netherlands

**Liming Li**

China

**Li-Na Chou**

Taiwan

**Lina Santaguida**

Canada

**Linda Douw**

Netherlands

**Linda Liebenberg**

Canada

**Linda Sharp**

Ireland

**Line Kjeldgaard Pedersen**

USA

**Lisa Chan**

USA

**Lisa Gold**

Australia

**Lisa Wintner**

Austria

**Lizzette Gómez-De-Regil**

Mexico

**L P Xiong**

China

**L Q Huang**

USA

**Luciana Scalone**

Italy

**Lucy Yardley**

UK

**Luis Rajmil**

Spain

**Luka Vučemilo**

USA

**L W Lam**

Hong Kong

**Lynne Murray**

UK

**M Abu-Helalah**

UK

**M C Yang**

Taiwan

**M Fokkema**

Netherlands

**M Joseph Sirgy**

USA

**M Khalil**

Egypt

**M Nunez**

Spain

**M O Owolabi**

Nigeria

**M Schuler**

Denmark

**M Subramaniam**

Singapore

**M Vuković**

USA

**M Younis**

USA

**M F Janssen**

Netherlands

**M A Abolfotouh**

Saudi Arabia

**Maeve Kerr**

UK

**Mahdi Najafi**

Iran

**Maiko Yagi**

Japan

**Makif Sariyildiz**

USA

**Man Hung**

USA

**Mansur Kutlubaev**

Russian Federation

**Manu Mathur**

India

**Manuel Bravo**

Spain

**Marcel Dijkers**

USA

**Marguerite Schneider**

South Africa

**Maria Crespo**

Spain

**Maria Teresa Rodriguez**

Spain

**Marie Preau**

Germany

**Mariko Naito**

Japan

**Marina Blanco-Aparicio**

Spain

**Marit Graue**

Norway

**Marita Rohr Inglehart**

USA

**Mark Harris**

Australia

**Mark Oppe**

Netherlands

**Mark Oremus**

Canada

**Martin Taphoorn**

Netherlands

**Maryam Hami**

Iran

**Marzieh Araban**

Iran

**Masahito Fushimi**

Japan

**Matt Stiefel**

USA

**Matthew Goldenberg**

USA

**Matthew HR Little**

USA

**Matthew Howren**

USA

**Matthew Rivara**

USA

**Matthew Zack**

USA

**Matthias Schneider**

Germany

**Maureen A Mathews**

USA

**Maureen Mcquestion**

Canada

**Mayssoon Dashash**

Syrian Arab Republic

**Megan Edgelow**

Canada

**Melissa Hyde**

Australia

**Meng-Hsing Wu**

Taiwan

**Meriel Jenney**

UK

**Michaael Clark**

USA

**Michael Brannick**

USA

**Michael Hyland**

UK

**Michael Jonathan Li**

USA

**Michal Farkowski**

Poland

**Michal Shteinberg**

Israel

**Michel Henry-Amar**

France

**Michelle Bovin**

USA

**Michelle Mocarski**

USA

**Miguel Maldonado Fernandez**

Spain

**Mihir Gandi**

Singapore

**Mika Miyashita**

Japan

**Mike Horton**

UK

**Mike John**

USA

**Mikkel Bek Clausen**

Denmark

**Min Hui Huang**

USA

**Minjeong Jeon**

USA

**Miriam Marks**

Switzerland

**Mirna Barbosa**

Brazil

**M J Chi**

Taiwan

**M N Aung**

Japan

**Mohammad Parnianpour**

Iran

**Mohammad Reza Hadian**

Iran

**Mohd Masood**

Malaysia

**Mojgan Karbakhsh**

Iran

**Molly Tanenbaum**

USA

**Mona Srivastava**

USA

**Monika Bullinger**

Germany

**Muirne Caitlin Shonagh Paap**

Netherlands

**Mun Chieng Tan**

New Zealand

**Murat Boysan**

USA

**Myzoon Ali**

UK

**N Papanas**

Greece

**Nadia Akseer**

Canada

**Nagwa Hasanin**

Egypt

**Nan Luo**

Singapore

**Natasha Noble**

Australia

**Nazan Bilgel**

Turkey

**N D Emerson**

USA

**Neeraj Chawla**

USA

**N G Mahtadi**

Canada

**Nicholas Pajewski**

USA

**Nicolas Lesouef**

USA

**Nicole Baumann**

UK

**Nicole Smith**

USA

**Nicolo Martinelli**

Italy

**Nikos Zourbanos**

Greece

**Nina Deng**

USA

**Nitin Sharma**

USA

**O D Osunde**

UK

**Ola Rolfson**

Sweden

**Olatz Garin**

Spain

**P Sakthong**

USA

**Pablo Martinez Martin**

Spain

**Padraig Dixon**

UK

**Paola Aceto**

USA

**Paola Bertapelle**

USA

**Paola Mosconi**

Italy

**Paolo Angelo Cortesi**

Italy

**Patricia Braun**

USA

**Patricia Poulin**

Canada

**Paul Jones**

UK

**Paul Veugelers**

Canada

**Pauline Lai**

Malaysia

**Paulo Martins-Junior**

Brazil

**Pavel Chernyshov**

USA

**P C Wu**

USA

**Pei Wang**

Singapore

**Pei-Xi Wang**

China

**Peter Fayers**

UK

**Peter Makai**

Netherlands

**Peter Robinson**

UK

**Peter Sand**

Sweden

**Phaedra Corso**

USA

**Pilar Cabanas-Grandío**

Spain

**Prafulla Kumar Swain**

India

**Priya Prasad**

USA

**Przemyslaw Paradowski**

Poland

**Qiuyue Zhong**

USA

**R Chiba**

Japan

**R De Miguel-Ibáñez**

Spain

**R Rosato**

Italy

**R Sutton**

UK

**Rachel Koelmeyer**

Australia

**Rachel Markwald**

USA

**Rafael Mesquita**

Netherlands

**Rahul Khanna**

USA

**Rahul Malhotra**

Singapore

**Ralph Crott**

Belgium

**Ramiro Verissimo**

Portugal

**Raquel Conceição Ferreira**

Brazil

**Reza Amini**

USA

**Rezzan Gunaydin**

Turkey

**Ricardo De Lima Zollner**

Brazil

**Richard Burns**

Australia

**Richard E Burney**

USA

**Richard Norman**

Australia

**Rizwana Roomaney**

USA

**Robert Heimer**

USA

**Robert Klaassen**

Canada

**Robert Ofenloch**

Germany

**Robert Perry**

USA

**Robert Wirth**

USA

**Roberta Ciampichini**

Italy

**Roberta Gualtierotti**

Italy

**Roberto Elosua**

Spain

**Rocio Barrios**

Spain

**Rocìo Martìn-Valero**

Spain

**Rod S Taylor**

UK

**Roger Keller Celeste**

Brazil

**Ron Hays**

USA

**Ronan Factora**

USA

**Rosalie Gorter**

Netherlands

**Roxanne Bainbridge**

Australia

**Roy G Elbers**

Netherlands

**Rui Campos**

Portugal

**Rui Viana**

Portugal

**S Helms**

USA

**S Persic**

Croatia

**S Simsek**

Netherlands

**Saad Bindawas**

Saudi Arabia

**Sadeq Ali Al-Maweri**

Yemen

**Saharnaz Nedjat**

Iran

**Sakurai Keiko**

Japan

**Sal Arlandis**

Spain

**Samer Kharroubi**

UK

**Sandrine Dabakuyo**

France

**Sangchoon Jeon**

USA

**Sara Conti**

Italy

**Sarah Stewart-Brown**

UK

**Sarina Muhamad Noor**

Malaysia

**Sasja Schepers**

Netherlands

**Saskia Weldam**

Netherlands

**Sawitri Assanangkornchai**

Thailand

**Seana Gall**

Australia

**Selma Dertlioglu**

USA

**Serena Ng**

Hong Kong

**Sergei Gerasimov**

USA

**Sergio Gonzalez**

Chile

**Shadiya Mohamed Saleh Baqutayan**

Malaysia

**Shariff-Ghazali Sazlina**

Malaysia

**Sharon Kirkpatrick**

Canada

**Sherry Taylor**

Canada

**Shivani Mhatre**

USA

**Shu-Ping Chen**

Canada

**S J Coons**

USA

**S J Wheelwright**

UK

**S L Carroll**

Canada

**Somsak Tiamkao**

Thailand

**Sonia Eremenco**

USA

**Sonia Quintao**

USA

**Soo-Jeong Lee**

USA

**Soonhee Roh**

USA

**Staffan Janson**

Sweden

**Stefan Cano**

UK

**Stefan Koeberich**

Germany

**Stefano Fagiuoli**

Italy

**Stephan Arndt**

USA

**Stephanie Sohl**

USA

**Stephen Hill**

Canada

**Stephen Jivraj**

UK

**Stephen Walters**

UK

**Sudaduang Krisdapong**

Thailand

**Sumit Thakar**

India

**Susan Griffey**

USA

**Susan Mahan**

USA

**Susan Parekh**

UK

**Su-Yun Li**

China

**Suzanne Richards**

UK

**T Borneman**

USA

**T Sugiyama**

Japan

**Tacjana Pressler**

USA

**Tais Barbosa**

Brazil

**Taís De Campos Moreira**

Brazil

**Tara Albrecht**

USA

**Tatjana Schnell**

Brazil

**Tatsunori Ikemoto**

Japan

**Theresa Keegan**

USA

**Thirumagal Kanagasabai**

Canada

**Thomas Dorner**

Austria

**Thomas Jozefiak**

Norway

**Thomas Reiberger**

Austria

**Thorsten Meyer**

Germany

**Tiffany Gill**

Australia

**Tim Gomersall**

UK

**Tm Rasekaba**

Australia

**Tony Szu-Hsien Lee**

Taiwan

**Tor-Ivar Karlsen**

Norway

**Toru Oga**

Japan

**Toscani M**

USA

**Tosin Popoola**

Canada

**Tracey Young**

UK

**Trude Reinfjell**

USA

**Tyler Renshaw**

USA

**Ulla Svantesson**

Sweden

**Urho M Kujala**

Finland

**Uta Kiltz**

Denmark

**Uwe Konerding**

Germany

**V A Lachanas**

USA

**V Bochkezanian**

Australia

**Vahe Khachadourian**

Armenia

**Valeria Mercadante**

UK

**Valerie Block**

USA

**Valerie Seror**

France

**Vedantam Rajshekhar**

India

**Veena Joshi**

India

**Victor Janine**

USA

**Vincenza Capone**

Italy

**Viviana Wuthrich**

Australia

**Vladisav Stefanovic**

Serbia and Montenegro

**Wai Kwong Tang**

Hong Kong

**Walter Wodchis**

Canada

**Wantanee Kulpeng**

Thailand

**Wei Huang**

USA

**Wenjie Duan**

China

**Wenru Wang**

Singapore

**Weon Young Lee**

Korea, Republic Of

**Wilfred Lau**

Hong Kong

**Wilfried Schupp**

Germany

**William W Thompson**

USA

**Wimalin Rimpeekool**

Australia

**Wiraphol Phimarn**

USA

**Wolfgang Greiner**

Germany

**Y Chang**

Taiwan

**Y Griep**

Belgium

**Yan Wu**

China

**Yang-Kun Chen**

USA

**Yaping Jin**

Canada

**Yaw-Wen Chang**

China

**Y B Zhu**

China

**Ying Liang**

China

**Ying Ying Leung**

Singapore

**Young-Jin Lim**

Korea, Republic Of

**Yu Sun**

USA

**Yuan Pang Wang**

Brazil

**Yura Loscalzo**

Italy

**Yutaka Matsuoka**

Japan

**Zahra Bagheri**

Iran

**Zeinab Jannoo**

USA

**K N Zhou**

China

**Zunyou Wu**

China

